# ZnCl_2_ treatment improves nutrient quality and Zn accumulation in peanut seeds and sprouts

**DOI:** 10.1038/s41598-020-59434-0

**Published:** 2020-02-11

**Authors:** Kai Zhao, Chengyin Zhao, Mengli Yang, Dongmei Yin

**Affiliations:** 1 College of Agronomy, Henan Agricultural University, Zhengzhou, 450002 China; 2 Life Science College, Luoyang Normal University, Luoyang, Henan 471934 China

**Keywords:** Plant sciences, Environmental sciences

## Abstract

Peanut is a popular food due to its high nutrient content. The effects of ZnCl_2_ on peanut seed germination, fatty acid and sugar contents, vitamin biosynthesis, antioxidant content, and Zn assimilation were evaluated in this study. Treatment with ZnCl_2_ significantly improved the germination rate, enhanced reactive oxygen species production and reduced the content of total fatty acids in peanut seed and sprout. However, ZnCl_2_ treatment did not reduce total sugar or total protein relative to the control. Germination promoted the biosynthesis of phenolics and resveratrol and increased the antioxidant capacity, as evaluated by Fe^3+^ reducing power and 2,2-diphenyl-1-picrylhydrazyl radical scavenging ability, especially under Zn stress conditions. The vitamin content decreased in the following order among treatments: germinated seeds with ZnCl_2_ treatment > germinated seeds without ZnCl_2_ treatment > dormant seeds. Interestingly, Zn content was approximately five times higher in the germinated ZnCl_2_-treated seeds compared to in the untreated germinated seeds and the dormant seeds. The results of this study provide a new method for producing healthy foods with enhanced vitamin content and antioxidant capacity.

## Introduction

Healthy and low-calorie diets are currently welcomed by consumers, which has led to an increase in the consumption of functional foods^1–4^. Foods with low levels of fatty acids (FAs) and sugar are expected to reduce energy production and benefit the health of consumers^5,6^.

Peanuts have a high content of nutrients, including plant protein, dietary fibre, unsaturated FAs, β-vitamins, vitamin E, Mg and numerous bioactive substances (e.g., flavonoids, resveratrol and plant sterols)^7,8^. Many overweight individuals avoid peanut consumption due to its high calorie content^9^. Peanut intake is thought to lead to weight gain and an increase in body mass index^10,11^.

A large amount of energy and resources are consumed during seed germination^12^, while a large amount of antioxidant substances are synthesized during this process^13,14^. Published data show that germination enhances the phenolic content and total antioxidant capacity (TAC) in many kinds of seeds^13^. In addition, abiotic stress treatment can enhance the content of antioxidant compounds in germinated seeds more than in dormant seeds^15,16^. For example, moderate concentrations of NaCl were found to enhance the biosynthesis of phenolics in radish sprouts^15^.

While heavy metals in food can have negative effects on human health^17^, certain heavy metals such as Zn, Fe and Cu are indispensable for human health at trace levels^18–20^. Among these beneficial elements, Zn is vital for human beings and particularly child development^21,22^. Although many efforts have been made to supplement foods with these beneficial elements, the results remain unsatisfactory^19^.

In this study, peanut seeds were soaked in ZnCl_2_ solution during the germination process. The contents of FAs, sugars, and vitamins along with the antioxidant capacities were compared between the dormant seeds and germinated seeds and sprouts under favourable and Zn stress conditions with the goal of answering two main questions: (1) Can Zn addition affect the nutrient content and antioxidant capacity of germinated peanut seeds and sprouts? (2) Can germination significantly improve Zn assimilation in germinated peanut seeds and sprouts? The results of this study provide a better understanding of how seed germination affects seed nutrient quality from the perspective of human health.

## Results

### Effects of Zn treatment on peanut seed germination, sprout growth, superoxide production and hydrogen peroxide accumulation

**Figure 1:**
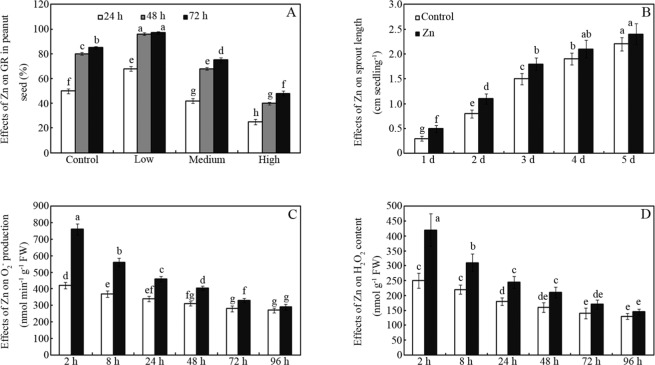
Effects of ZnCl_2_ treatment on germination rate (**A**), sprout length (**B**), O_2_^−^ production (**C**) and H_2_O_2_ content (**D**) in peanut seeds and sprouts. The bars represent standard deviations of means (*n* = 3). Means followed by the same letter are not significantly different (*p* < 0.05) among treatments.

As shown in Fig. 1A, the germination ability of peanut seeds was significantly affected by ZnCl_2_. Compared to the control, treatment with a low concentration (20 mM) of ZnCl_2_ significantly increased the germination rate (GR) by 36%, 20% and 14% after imbibition for 24, 48 and 72 h, respectively (Fig. 1A; *p* < 0.05). However, high concentrations of ZnCl_2_ (100 and 200 mM) significantly decreased the GR by 12% and 44%, respectively, after imbibition for 72 h (Fig. 1A; *p* < 0.05). Thus, the Zn treatment concentration in subsequent experiments was 20 mM. Similarly, Zn treatment at 20 mM significantly enhanced sprout growth, particularly in the early stage (Fig. 1B). Compared to the water control, sprout length was increased by approximately 67%, 38%, 20%, 11% and 10% after 1, 2, 3, 4, and 5 d, respectively (Fig. 1B; *p* < 0.05).

Treatment with Zn also significantly enhanced superoxide (O_2_^−^) production and hydrogen peroxide (H_2_O_2_) accumulation in germinated seeds and sprouts compared to the water control. Zn increased O_2_^−^ production by 81%, 51%, 35%, 31%, 18% and 7% after seed imbibition for 2, 8, 24, 48, 72 and 96 h, respectively (Fig. 1C; *p* < 0.05). Similar results were also observed for H_2_O_2_ accumulation in germinated seeds and sprouts after Zn treatment; for example, the H_2_O_2_ content was increased by approximately 68% and 41% compared to the control after Zn treatment for 2 and 8 h, respectively (Fig. 1D; *p* < 0.05).

### Effects of Zn treatment on the contents of total FA, total sugar and total protein

**Figure 2:**
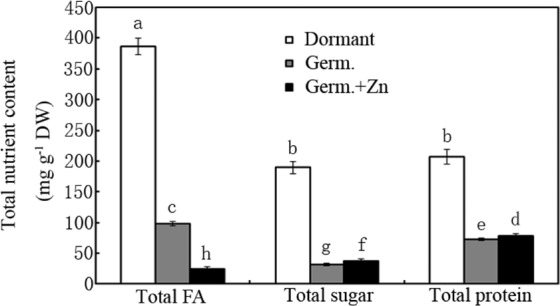
Effects of ZnCl_2_ treatment on total FA, total sugar and total protein contents in peanut seeds and sprouts. Bars represent standard deviations of means (*n* = 3). Means followed by the same letter are not significantly different (*p* < 0.05) among treatments.

The effects of ZnCl_2_ treatment (20 mM) on the contents of nutrients (total FA, total sugar and total protein) were evaluated. As shown in Fig. 2, total FA, total sugar and total protein were respectively reduced by 75%, 83% and 65% in the germinated peanut seeds and sprouts compared to the dormant seeds (*p* < 0.05). Compared to the water control, ZnCl_2_ treatment decreased the content of total FA in germinated peanut seeds and sprout by 74% and increased the contents of total sugar and total protein by 20% and 9%, respectively (Fig. 2; *p* < 0.05).

### Effects of Zn treatment on FA and sugar composition

Seven main FAs were detected in dormant and germinated peanut seeds and sprouts (Table 1), and their contents decreased in the following order: linolic acid > oleic acid > palmic acid > stearic acid > behenic acid > arachic acid > lignoceric acid. Compared to the dormant seeds, the contents of all these FAs decreased after germination. For example, the content of linolic acid was reduced by approximately 74% and 95% after germination in the water control and Zn-treated group, respectively (Table 1; *p* < 0.05).

**Table 1:** Effects of ZnCl_2_ treatment on FA composition in peanut seeds and sprouts Contents of different FAs in dormant and germinated (control and Zn treatment) peanut seeds and sprouts (g kg^−1^ DW). Means followed by the same letter are not significantly different (*p* < 0.05) among different treatments in each row. Three replicates were analysed for each treatment.

	Dormant seeds	Water control seeds	ZnCl_2_-treated seeds
Oleic acid (18:1)	44.5 ± 1.1a	9.2 ± 0.7b	4.5 ± 0.6c
Linolic acid (18:2)	51.2 ± 1.3a	13.3 ± 0.5b	2.2 ± 0.4c
Palmic acid (16:0)	16.9 ± 0.8a	1.5 ± 0.2b	1.1 ± 0.2b
Stearic acid (18:0)	4.3 ± 0.7a	1.2 ± 0.2b	0.9 ± 0.1b
Behenic acid (22:0)	2.5 ± 0.3a	0.5 ± 0.1b	0.4 ± 0.1b
Arachic acid (20:0)	1.7 ± 0.2a	0.7 ± 0.1b	0.5 ± 0.1b
Lignoceric acid (24:0)	1.1 ± 0.2a	0.3 ± 0.1b	0.2 ± 0.1b

Four main non-structural sugars were detected in dormant and germinated peanut seeds and sprouts (Table 2), and the contents decreased in the following order: starch > sucrose > glucose > fructose. Germination reduced the starch and sucrose contents but enhanced the glucose and fructose contents. For example, the sucrose and starch contents were respectively decreased by approximately 75% and 82% in ZnCl_2_-treated group compared to the dormant seeds (Table 2; *p* < 0.05).

**Table 2:** Effects of ZnCl_2_ treatment on sugar composition in peanut seeds and sprouts Contents of different sugars in dormant and germinated (control and Zn treatment) peanut seeds and sprouts. Means followed by the same letter are not significantly different (*p* < 0.05) among different treatments in each row. Three replicates were analysed for each treatment.

	Dormant seeds	Water control seeds	ZnCl_2_-treated seeds
Glucose (mg·g^−1^ FW)	0.7 ± 0.1c	1.5 ± 0.1b	1.8 ± 0.2a
Fructose (mg·g^−1^ FW)	0.3 ± 0.1c	0.7 ± 0.1b	0.9 ± 0.2a
Sucrose (mg·g^−1^ FW)	9.2 ± 0.9a	1.4 ± 0.2c	2.3 ± 0.1b
Starch (mg·g^−1^ FW)	74.4 ± 3.2a	21.3 ± 1.7b	13.1 ± 2.5c

### Effects of Zn treatment on antioxidant capacity

**Figure 3:**
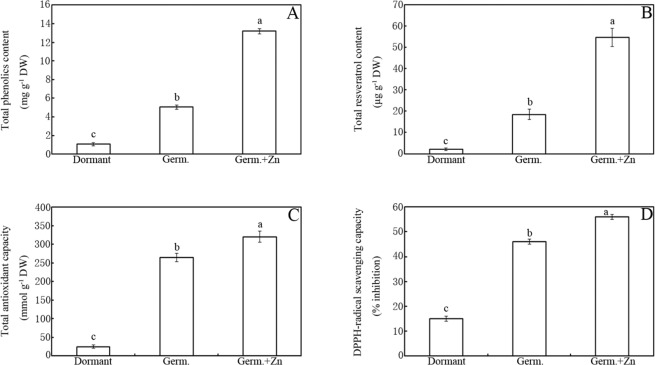
Effects of ZnCl_2_ treatment on total phenolic content (**A**), resveratrol content (**B**), TAC (**C**) and DPPH-radical scavenging capacity (**D**) in peanut seeds and sprouts. Bars represent standard deviations of means (*n* = 3). Means followed by the same letter are not significantly different (*p* < 0.05) among treatments.

Compared to the dormant seeds, germination increased the antioxidant (e.g., total phenolics and resveratrol) content and antioxidant capacity, as evaluated by Fe^3+^ reducing power/TAC and 2,2-diphenyl-1-picrylhydrazyl (DPPH) radical scavenging capacity, and this effect was further enhanced by ZnCl_2_ treatment (Fig. 3). For example, total phenolic content was approximately 376% and 1145% higher in germinated peanut seeds and sprouts under favourable and Zn stress conditions, respectively, compared to in the dormant seeds (Fig. 3A; *p* < 0.05). Similarly, the resveratrol content was approximately eight and 25 times higher in the germinated seeds and sprouts in the water control and Zn-treated groups, respectively, compared to in the dormant seeds (Fig. 3B). The TAC content was approximately 9.6 times higher (water control) and 11.8 times higher (ZnCl_2_ treatment) in the germinated seeds and sprouts compared to in the dormant seeds (Fig. 3C; *p* < 0.05). ZnCl_2_ treatment enhanced the DPPH-radical scavenging capacity by 22% compared to the water control (Fig. 3D; *p* < 0.05).

### Effects of Zn treatment on vitamin and Zn contents

ZnCl_2_ treatment enhanced the biosynthesis of all studied vitamins with the exception of vitamin B_1_ (Table 3). The contents of vitamins A, B_2_, C, and E were increased by approximately 11.9, 7.5, 18, and 3.7 times in the germinated ZnCl_2_-treated seeds and sprouts compared to the dormant seeds (Table 3; *p* < 0.05). In contrast, the content of vitamin B_1_ decreased by approximately 45% and 36% after germination under favourable and Zn stress conditions, respectively, compared to the dormant seeds (Table 3; *p* < 0.05). Finally, the Zn content was approximately five times higher in the germinated ZnCl_2_-treated seeds compared to in the dormant seeds (Table 3; *p* < 0.05).

**Table 3:** Effects of ZnCl_2_ treatment on vitamin and Zn contents in peanut seeds and sprouts Vitamin and Zn contents in dormant and germinated (control and Zn treatment) peanut seeds and sprouts. Means followed by the same letter are not significantly different (*p* < 0.05) among different treatments in each row. Three replicates were analysed for each treatment.

	Dormant seeds	Water control seeds	ZnCl_2_-treated seeds
Vitamin A (μg·g^−1^ DW)	12 ± 2a	80 ± 6b	155 ± 12c
Vitamin B_1_ (μg·g^−1^ DW)	3100 ± 58a	1690 ± 82b	1990 ± 35c
Vitamin B_2_ (μg·g^−1^ DW)	199 ± 23a	1010 ± 37b	1699 ± 27c
Vitamin C (μg·g^−1^ DW)	20 ± 2a	250 ± 22b	380 ± 15c
Vitamin E (μg·g^−1^ DW)	29 ± 4a	82 ± 2b	136 ± 3c
Zn element (μg·g^−1^ DW)	18 ± 2a	21 ± 1a	127 ± 5b

## Discussion

Many studies have been carried out on the effects of germination on antioxidant contents and TAC in seeds^14,15,23^. Less data are available to show the correlations between nutrients (e.g., FAs and sugar) consumption and antioxidant biosynthesis during seed germination, especially under abiotic stress conditions.

In this study, the FA, sugar, vitamin, antioxidant, and Zn contents were compared between dormant and germinated peanut seeds and sprouts. As shown in Fig. 1, treatment with high concentrations of ZnCl_2_ (e.g., 100 and 200 mM) inhibited peanut seed germination compared to the water control. However, treatment with a low concentration of ZnCl_2_ (20 mM) significantly increased the seed GR compared to the water control (Fig. 1A). Thus, moderate Zn treatment can improve peanut seed germination. Interestingly, Zn treatment also improved seedling/sprout growth (Fig. 1B). We speculate that this phenomenon may be associated with Zn-mediated reactive oxygen species (ROS), which can accelerate seed germination and plant growth^24–26^. Thus, the O_2_^−^ production and H_2_O_2_ content were investigated. Zn addition markedly increased O_2_^−^ production and H_2_O_2_ content, particularly at the early stage of germination (Fig. 1C,D). Thus, the ability of Zn to promote seed germination and sprout growth is likely related to its effects on ROS production.

Peanut seeds can preserve a high percentage of energy compounds, including fat and sugar^7,8^. The effects germination with and without moderate Zn treatment on the total FA, total sugar and total protein contents were evaluated herein (Fig. 2). Germination significantly decreased the contents of total FA, total sugar and total protein compared to the dormant seeds, regardless of whether ZnCl_2_ was applied. The main types of FAs (e.g., oleic acid and linolic acid) and sugars (e.g., starch and sucrose) were further investigated (Tables 1 and 2). The main FAs were oleic acid, linolic acid, and palmic acid, especially in dormant peanut seed (Table 1). The contents of these FAs were decreased significantly by germination, particularly after ZnCl_2_ treatment (Table 1). The contents of non-structural carbohydrates (e.g., starch) were also reduced in the germinated seeds and sprouts, especially under Zn treatment conditions (Table 2). In contrast, the sucrose content was reduced more significantly in the germinated seeds without Zn treatment than in those with Zn treatment (Table 2). Furthermore, the contents of glucose and fructose increased after seed germination (Table 2). These results demonstrate that the changes in non-structural carbohydrates during germination depend on the type of sugar, which raises another interesting question: Why does Zn treatment further accelerate the degradation of FAs and starches during germination compared to the water control? One plausible explanation is that Zn-mediated ROS promoted seedling growth and metabolic activity, requiring more energy (e.g., adenosine triphosphate) to be produced via FA and starch degradation.

High contents of antioxidants are indispensable for deterring seed ageing^27,28^. Thus, oxidative stress is required for seed dormancy release^26,29^. It seems that a large amount of antioxidants may be consumed after seed germination. To confirm this speculation, the antioxidant contents (e.g., total phenolics and resveratrol) and antioxidant capacity were compared between the dormant and germinated seeds and sprouts (Fig. 3). Germination was found to significantly enhance antioxidant content and capacity (Fig. 3), in accordance with a previous report^30^ showing that seed germination can significantly enhance the accumulation of total phenolics and resveratrol in sprouts. Interestingly, treatment with 20 mM ZnCl_2_ further enhanced the antioxidant content and antioxidant capacity (Fig. 3). Compared to the water control, ZnCl_2_ treatment increased the total phenolic and resveratrol contents in germinated peanut seeds and sprouts by approximately 2.6 and 3 times, respectively (Fig. 3A,B). Similarly, a past study found that abiotic stress (ultraviolet radiation and H_2_O_2_) significantly increased resveratrol biosynthesis in peanut plant^31^. Accordingly, greater TAC and higher DPPH radical scavenging were observed in the ZnCl_2_-treated seeds compared to in the water control group (Fig. 3C,D). This effect might be explained by Zn-mediated ROS, which can promote antioxidant biosynthesis in plants^32^.

Seed germination was found to increase the contents of vitamins A, B_1_, B_2_, C and E compared to the dormant seeds, especially under Zn treatment (Table 3). Interestingly, certain vitamins (e.g., vitamins C and E) are known antioxidants^32^. This suggests that high quantities of antioxidant and metabolite molecules are required for peanut seed germination, especially after Zn treatment. The findings show that ZnCl_2_ treatment not only reduced FA accumulation, but also enhanced the antioxidant, vitamin and Zn contents in germinated peanut seeds and sprouts. Zn supplementation can be extended to other seed germination-derived foods. Thus, Zn treatment can be used to produce healthy foods in the future.

In conclusion, seed germination reduced the total FA, total protein and total sugar contents while enhancing antioxidant and vitamin contents in peanut seeds compared to the dormant seeds. Treatment with a low concentration of ZnCl_2_ improved seed germination, further reduced the contents of energy-storing compounds, further enhanced antioxidant content, and led to Zn assimilation. Thus, treatment with ZnCl_2_ offers a new way to increase the antioxidant capacity and Zn content during seed germination.

## Materials and Methods

### Seed treatment

Peanut seeds (*kainong 70*; *Arachis hypogaea* Linn.) were obtained from a seed distributor in Zhengzhou, China. The seeds were sown in plastic boxes and placed in a seed germinator at room temperature. Germination trials were conducted in plastic boxes equipped with 4-cm-deep sand with a particle diameter of approximately 0.5 mm. The sand was moistened with distilled water or water containing different concentrations of ZnCl_2_. Before seed germination, half of the control seeds were moistened with distilled water for 30 min. After treatment, the seeds were transferred to plastic boxes.

This experiment was divided into two treatment groups. In group 1, four concentrations of ZnCl_2_ (0, 20, 100 and 200 mM) were applied to the water-moistened seeds. In group 2, the Zn-treated peanut seeds were placed in ZnCl_2_-soaked sand for germination. All assays were replicated at least three times, and each replicate was carried out on 50 seeds. When the radicle emerging from the peanut seed reached a length of 1 cm, the seeds and sprouts were collected for subsequently assay.

### GR and sprout length assay

GRs were calculated as the percentage of germinated peanut seeds after sowing for different time periods^33,34^. Sprout length was measured using Vernier callipers.

### O_2_^−^ and H_2_O_2_ content assays

O_2_^−^ production was determined by monitoring nitrate formation from hydroxylamine in the presence of O_2_^−^ generators, as described by Elstner and Heupel^35^. Seed and sprout tissues were homogenized with liquid nitrogen in a chilled pestle and mortar with 1 mL of 65 mM Ki-phosphate buffer (pH 7.8). The homogenate was centrifuged at 5,000 × g for 10 min at 4 °C. Ki-phosphate buffer (0.45 mL; pH 7.8) and 10 mM hydroxylamine hydrochloride (50 μL) were added to the supernatant. After developing this mixture at room temperature for 20 min, 0.5 mL of the mixture was added to a solution (0.5 mL) containing 17 mM anaphthaleneamine at room temperature for 20 min. The mixture was vortexed and centrifuged at 1,500 × g for 5 min. The absorbance of the pink aqueous phase was then recorded at 530 nm. A standard curve of NO_2_^−^ was used to calculate the production rate of O_2_^−^ from the chemical reaction of hydroxylamine and O_2_^−^.

The H_2_O_2_ contents in treated peanut seed were determined using a previously reported method^36^. H_2_O_2_ production in peanut seeds and sprouts was determined using the oxidation xylenol orange assay, which is based on the oxidation of Fe^2+^ ions by peroxide followed by colorimetric detection of the reaction of Fe^3+^ with the sodium salt of xylenol orange. One millilitre of assay reagent (25 mM FeSO_4_ and 25 mM (NH_4_)_2_SO_4_ dissolved in 2.5 M H_2_SO_4_) was added to 100 mL of 125 μM xylenol orange and 100 mM sorbitol. The collected tissue was ground and centrifuged at 8000 × g for 5 min. The supernatant (100 μL) was added to 1 mL of xylenol orange reagent. After 30 min of incubation, the absorbance of the Fe^3+^–xylenol orange complex was recorded at 560 nm using a spectrophotometer.

### Assay for total phenolics content

The peanut seeds and sprouts were collected for the measurement of total phenolics. The content of total phenolics was determined using Folin–Ciocalteu reagent^37^. Two grams of sample was extracted for 2 h with 20 mL of 80% methanol containing 1% hydrochloric acid at 25 °C on an orbital shaker at 200 rpm. The mixture was centrifuged at 1000 × g for 20 min, and the supernatant was transferred to 100-mL vials. The supernatants were combined and used for the total phenolics assay. The extract (1 mL) was mixed with 7.5 mL of Folin–Ciocalteu reagent and allowed to stand at room temperature for 5 min. Next, 7.5 mL of 60 g·L^−1^ sodium bicarbonate solution was added to the mixture. After allowing to stand for 90 min at room temperature, the absorbance was recorded at 725 nm. The results are expressed as ferulic acid equivalents.

### Resveratrol extraction and assay

Resveratrol was determined using the method of Fettig and Hess with modification^38^. Peanut seeds and sprouts were protected from light during analysis. The free form of resveratrol was extracted by agitating the frozen sample powder in methanol for 16 h at 25 °C. Resveratrol was separated using a μBondapak C18 column and assayed with a fluorescence detector (excitation wavelength of 330 nm and emission wavelength of 374 nm). Samples from three replications were analysed.

### Antioxidant capacity assay

The peanut seeds and sprouts collected for antioxidant capacity assay were weighed and immediately frozen in liquid N_2_. Dry samples (1 g) were ground into a powder in liquid N_2_ using a mortar and pestle, transferred to 100 mL of 90% (w/v) methanol/water solution, and incubated at room temperature for 24 h in the dark. The extracts were filtered, the filtrates from each replicate were pooled, and the solvent was evaporated under vacuum at 45 °C using a rotary evaporator. The resulting crude extracts were stored in a desiccator at 4 °C until being analysed for TAC and ROS scavenging capacity.

TAC was evaluated by ferric reducing ability of plasma assay^39^. The DPPH radical scavenging method was used to evaluate the ROS scavenging capacity^40,41^. DPPH solution in methanol (50 μM) was freshly prepared, and 2.9 mL of this solution was mixed with 100 μL of extract. The samples were incubated for 30 min at 37 °C in a water bath, and the decrease in absorbance at 515 nm was measured (*A*_*E*_). The DPPH solution was used as a blank sample, and its absorbance was also measured (*A*_*B*_). The DPPH radical scavenging capacity was calculated as follows: percentage of inhibition = [(*A*_*B*_*−A*_*E*_)/*A*_*B*_] × 100%.

### Total FA and composition assay

Peanut seeds and sprouts were collected for total FA assay. The total FA content and composition were determined using the method of Andersen *et al*.^42^.

### Total sugar and non-structural carbohydrate assay

Peanut seeds and sprouts were collected for total sugar and non-structural carbohydrate assay. The levels of glucose, sucrose, fructose, starch (amyloglucosidase was used to hydrolyse starch) and total sugar were determined by high-performance liquid chromatography (HPLC)^43^. Peanut seed and sprout tissues (2 g) were fixed in 96% ethanol, and carbohydrates were extracted with 80% ethanol. The extracts were purified by a solid-phase extraction technique. Soluble carbohydrates (glucose, sucrose and fructose) were analysed using a Diasorb-130-Amin column (250 × 4 mm) packed with 6-µm-diameter particles. A refractometer was used as a detector, and an acetonitrile:water mixture (v:v = 70:30) was used as the eluent at a flow rate of 0.6 mL·min^−1^. The D-forms of fructose, glucose and sucrose were used as standards. The amount of soluble carbohydrates was calculated using the absolute calibration method.

### Vitamin and Zn assays

Peanut seeds and sprouts were collected for vitamin A, B_1_, B_2_, C and E determination using HPLC according to the method of Feliciano *et al*.^44^. The Zn content was assayed using the method of Deng *et al*.^41^ Zn-treated peanut seeds and sprouts were collected, washed, air-dried and ground into powder using a mortar and pestle. For Zn extraction, digestion tubes were acid-washed and dried. Each dried powdered sprout sample (1 g each) was added to a digestion tube followed by the addition of 10 mL of a mixture of HNO_3_, HClO_4_ and H_2_SO_4_ (volume ratio = 5:1:1) to each tube. After 12 h, the tubes were placed in a digestion block at 80 °C for approximately 1 h. The temperature was then increased slowly to 120 °C–130 °C. When digestion was complete, the solutions were cooled, filtered and diluted to 100 mL with doubly deionized water. The filtrates were assayed for Zn using atomic absorption spectrometry (Analyst 700, Perkin Elmer, USA).

### Data analysis

The experiments were conducted in a completely randomized design. Three replicates were analysed for each treatment. All data were analysed by Duncan’s multiple range test (*p* < 0.05) using SPSS 13.0 software.
